# High-throughput color determination of red raspberry puree and correlation of color parameters with total anthocyanins

**DOI:** 10.1186/s13007-024-01197-0

**Published:** 2024-05-30

**Authors:** Claudia Baldassi, Clover Lee, Michael Dossett, Simone D. Castellarin

**Affiliations:** 1 Wine Research Centre, Faculty of Land and Food Systems, The University of British Columbia, 2205 East Mall, Vancouver, BC V6T 1Z4 Canada; 2 BC Berry Cultivar Development Inc., Abbotsford, BC Canada

**Keywords:** *Rubus idaeus* L., Puree color, Tomato analyzer color test, Digital phenotyping, Colorimeter, Total anthocyanin content

## Abstract

**Background:**

Red raspberry fruit color is a key driver of consumer preference and a major target of breeding programs worldwide. Screening for fruit color typically involves the determination of anthocyanin content and/or the assessment of color through a colorimeter. However, both procedures are time-consuming when the analyses involve hundreds or thousands of samples. The objectives of this study were to develop a high-throughput method for red raspberry puree color measurement and to test the correlations between color parameters and total anthocyanin content. Color coordinates were collected with a colorimeter on 126 puree samples contained in Petri dishes and with the Tomato Analyzer Color Test (TACT) module to assess the same samples prepared in Petri dishes and in 96-well plates. An additional 425 samples were analyzed using only 96-well plates. Total anthocyanins were extracted from all 551 samples.

**Results:**

Regression models for L*, a*, b* measured with the colorimeter and TACT using Petri dishes were all significant (*p* < 0.001), but very consistent only for L* (*R*^*2*^ = 0.94). Significant (*p* < 0.001) and very consistent regressions (*R*^*2*^ = 0.94 for L* and b*, *R*^*2*^ = 0.93 for a*) were obtained for color parameters measured with TACT using Petri dishes and TACT using plates. Of the color parameters measured with the colorimeter, only L*, a*/b*, and hue significantly correlated with total anthocyanins (*p* < 0.05), but, except for L* (*R* = − 0.79), the correlations were weak (*R* = − 0.23 for a*/b* and *R* = 0.22 for hue). Conversely, all correlations with total anthocyanins and color parameters measured with TACT were significant (*p* < 0.001) and moderately strong (e.g., *R* = − 0.69 for L* and *R* = 0.55 for a*/b*). These values were indicative of darker colors as total anthocyanin content increased.

**Conclusions:**

While the colorimeter and TACT-based methods were not fully interchangeable, TACT better captured color differences among raspberry genotypes than the colorimeter. The correlations between color parameters measured with TACT and total anthocyanins were not strong enough to develop prediction models, yet the use of TACT with 96-well plates instead of Petri dishes would enable the high-throughput measurement of red raspberry puree color.

**Supplementary Information:**

The online version contains supplementary material available at 10.1186/s13007-024-01197-0.

## Background

Red raspberry (*Rubus idaeus* L.) is an economically important berry crop worldwide, whose production has increased over the past decade [1]. Mexico, California, and the Pacific Northwest (Oregon, Washington, British Columbia) are the major red raspberry producers in North America [1, 2]. Red raspberries are highly acclaimed for their sensory quality and putative health benefits [3–7]. Among quality characteristics, berry color is a key driver of market acceptability and consumer preference [8, 9], and therefore a trait of interest for breeding programs. Red raspberry fruit encompasses a wide array of colors, including red (with intensities ranging from very light to deep dark), orange, and yellow [9]; however, red fruited cultivars are the most common in commercial settings [10]. The fresh raspberry market asks for cultivars with bright light- or medium-red and non-darkening fruit, while the processing industry prefers cultivars with dark berries for most applications [11]. Anthocyanins, a group of phenolic compounds, are regarded as the major contributors to raspberry red fruit color [12, 13]. The most abundant anthocyanins in red raspberry are cyanidin-glycosides such as cyanidin-3-sophoroside, cyanidin-3-(2 g-glucosylrutinoside), cyanidin-3-glucoside, cyanidin-3-rutinoside, and pelargonidin-glycosides such as pelargonidin-3-sophoroside, pelargonidin-3-(2g-glucosylrutinoside), pelargonidin-3-glucoside, pelargonidin-3-rutinoside) [14–17]. A preponderance of cyanidin glycosides confers a deep red color to fruit, while a preponderance of pelargonidin glycosides determines an orange-red color [18]. Extraction of anthocyanins is achieved with simple protocols and commonly available solvents and supplies [19]. However, anthocyanin extraction generates hazardous waste and turns into a time-consuming and costly process when working with large sample volumes [19]. Non-destructive techniques based on Vis-NIR spectroscopy, spectral imaging, and computer vision have been developed to estimate anthocyanin content in small fruits [20–24], but such techniques often require expensive equipment and/or specialized knowledge [25], making it difficult to implement in breeding programs. In addition, Andrianjaka-Camps, Baumgartner [26] used IR spectroscopy to predict several quality traits of raspberry puree, but they could not obtain an accurate prediction model for total anthocyanins. Establishing a methodology to estimate anthocyanins in a quick and inexpensive way would optimize the screening of this fruit trait and greatly benefit raspberry breeding programs that produce and evaluate hundreds or thousands of new selections, with the aim of developing new cultivars with premium fruit color for the fresh and processed industry. The correlation between anthocyanin content (total anthocyanins and anthocyanin composition) and fruit color has been investigated in red raspberry but has produced contrasting results [16, 19, 27–29]. Standard procedures for fruit color assessment rely on the tristimulus colorimeter, an instrument that allows to measure color by simply placing objects on the instrument sensor eye. Nevertheless, colorimeters usually can measure only one sample at a time, also making color measurement a slow and tedious activity. Digital imaging paired with digital phenotyping software offers the opportunity to conduct color measurement at a much faster pace than the colorimeter. Tomato Analyzer (TA) is a software developed for high-throughput phenotyping of several fruit traits, including color, which can be assessed through the Tomato Analyzer Color Test (TACT) module [30–32]. The TACT module was developed using fresh tomato fruit and was validated by comparing color measurements of standard Munsell color plates collected with TACT and a colorimeter [31]. Since its development, the TACT has been used to assess fruit color of some commercial crops, including tomatoes [31, 33] and dates [34]. To the authors’ knowledge, the TACT has been implemented on fresh fruit only, without applications on fruit processed products, such as juices or purees. In experimental settings, raspberry puree or juice are frequently used instead of fresh fruit to assess fruit quality parameters such as soluble solids content, titratable acidity, pH, and color [16, 27], therefore making it convenient to use a high-throughput method to collect color information from pureed fruit. The objectives of this study were twofold: (i) to develop a protocol for high-throughput color analysis of red raspberry puree using TACT, and (ii) to determine whether TACT could offer advantages over the colorimeter in terms of estimation of total anthocyanin content in red raspberry puree.

## Methods

### Plant material

Fruit was harvested from red raspberry (*Rubus idaeus* L.) commercial cultivars and selections grown at the Agriculture and Agri-Food Canada (AAFC) Clearbrook sub-station in Abbotsford (British Columbia, Canada). A total of 551 genotypes were used in this study; these genotypes included both commercial cultivars and breeding program selections not being grown commercially. Raspberry plants were planted between 2000 and 2018, in rows 3.05 m apart and spaced 3.05 m apart within the row. Twenty-five berries per genotype were collected at commercial maturity (June through August) during the 2020 season. Fruit was frozen at − 20 °C immediately after harvest. During processing, fruit samples were allowed to thaw at room temperature for 2–3 h, then homogenized with a blender (NutriBullet 600 W, Nutribullet, Los Angeles, CA, USA) into a puree. Puree samples were collected in resealable plastic bags (one bag per genotype) and stored at − 20 °C until analysis.

### Extraction of total anthocyanins

Aliquots of frozen raspberry puree were poured in plastic cups and let thaw for one hour at room temperature. Puree aliquots (0.15 g) were weighed into 2 ml microcentrifuge tubes, in triplicates, and extracted with a 50% methanol and 1% formic acid solvent (2 × 1.5 mL) [35]. After solvent addition, samples were sonicated for 20 min and centrifuged for 10 min at 13,000 g and 4 °C. Extracts from the same genotype were combined into new tubes. Samples were immediately assessed for total anthocyanins or stored at − 80 °C and analyzed 12 h after extraction.

### Determination of total anthocyanins

Total anthocyanin content was determined through the pH differential method [36–38]. Samples were diluted 1:10 in water solutions at pH 1 (prepared using NaCl and HCl) and pH 4.5 (prepared using KCl and HCl) in 96-well clear plates. The absorbance was measured at 510 and 700 nm with a plate reader (Infinite® 200 PRO, Tecan Austria GmbH, Grödig, Austria). Total anthocyanins were quantified as cyanidin-3-glucoside (C3G) equivalents (mg/L) based on a C3G calibration curve. The following formula was used to determine total anthocyanins (mg/100 g fresh weight, FW):

$$\text{Total}\;\text{anthocyanins}\; (\text{mg}/100 \text{g}\,\text{FW}) =\left(\frac{TAC}{}\times \frac{\frac{PEV}{\text{1,000}}}{FW}\right)\times 100$$

Where TAC = total anthocyanin content expressed as C3G equivalents (mg/L), PEV = phenolic extract volume (mL), 1,000 = factor for converting mL to L, FW = fresh weight (g), and 100 = factor for expressing the value per 100 g.

### Color determination with the colorimeter and Tomato Analyzer

Pictures of the experimental setup for color analyses with the colorimeter and Tomato Analyzer are provided in Additional file 1: Fig. S1.

#### Sample preparation

Frozen raspberry puree aliquots were poured in plastic cups and kept at room temperature until completely thawed. Puree aliquots of 4 mL and 280 µL were pipetted, respectively into 3.5-cm Petri dishes and in 96-well clear plates, in triplicates. The volume 4 mL was chosen after comparing the effect of different puree volumes on color coordinates L*, a*, b* collected with the colorimeter and Tomato Analyzer, as such volume determined on average the lowest variability in all the color parameters using either method (Additional file 2: Fig. S2, Additional file 3: Fig. S3, Additional file 4: Table S1, Additional file 5: Table S2). The volume 280 µL corresponded to nearly the maximum capacity (300 µL) of the plate wells. A total of 126 samples were analyzed using Petri dishes and 96-well plates, and another 425 samples were measured using exclusively 96-well plates.

#### Colorimeter analysis

The color of samples contained in Petri dishes (126 genotypes × 3 reps = 378 samples) was measured with a Hunter Lab colorimeter (LabScan XE, Reston, VA, USA), equipped with a 3-cm sensor eye and using the 1976 CIELab color space (illuminant D65, observer 10°). The colorimeter was calibrated using standard white and black reflective plates. The following color parameters were collected: L* as the indicator for lightness, spanning 0 (black) to 100 (white); a* (–128 to 127) as the indicator for green (negative values) to red (positive values); b* (–128 to 127) as the indicator for blue (negative values) to yellow (positive values). The parameters Chroma and hue, defined respectively as the color intensity and the combination of basic colors (red, blue, yellow), were derived as follows: Chroma = sqrt(a*^2^+b*^2^); hue = arctan(b*/a*). The ratio a*/b* was also computed. Blank values were obtained by measuring three empty Petri dishes and blanks’ L*, a*, b* values were deducted from samples’ L*, a*, b* values.

#### Tomato analyzer analysis

To allow equal spacing of Petri dishes on the scanner bed, a black construction paper mask was created by cutting ∼ 2.5-cm circles about 1 cm apart from each other. The mask was accommodated on a flatbed scanner (Perfection V600 Photo, Epson, Suwa, Japan) and the Petri dishes were placed on top of the carved circles. Ninety-six-well plates were placed directly on the scanner bed. Scans were acquired at 600 dpi and by covering the scanner with a cardboard box. All images were saved in jpg format and processed in FireAlpaca v. 2.11.17 on a Windows 11 Home v. 23H2 computer, to replace the original background with a black background (Additional file 6: Fig. S4). The final resolution of the images after editing was maintained at 600 dpi. Image analysis was performed with Tomato Analyzer (TA) v. 4.0 on a Windows 8.1 with Bing computer. Tomato Analyzer calibration was done using a color checker (ColorChecker Passport Photo 2, Calibrite LLC, Wilmington, DE, USA) and setting illuminant D65, observer 10° of the CIELab color space. The Color Test function of TA (TACT) was used to obtain L*, a*, b*, hue, and Chroma of all samples. Parameters a* and b* were used to compute the a*/b* ratio.

#### Color category definition

**Fig. 1:**
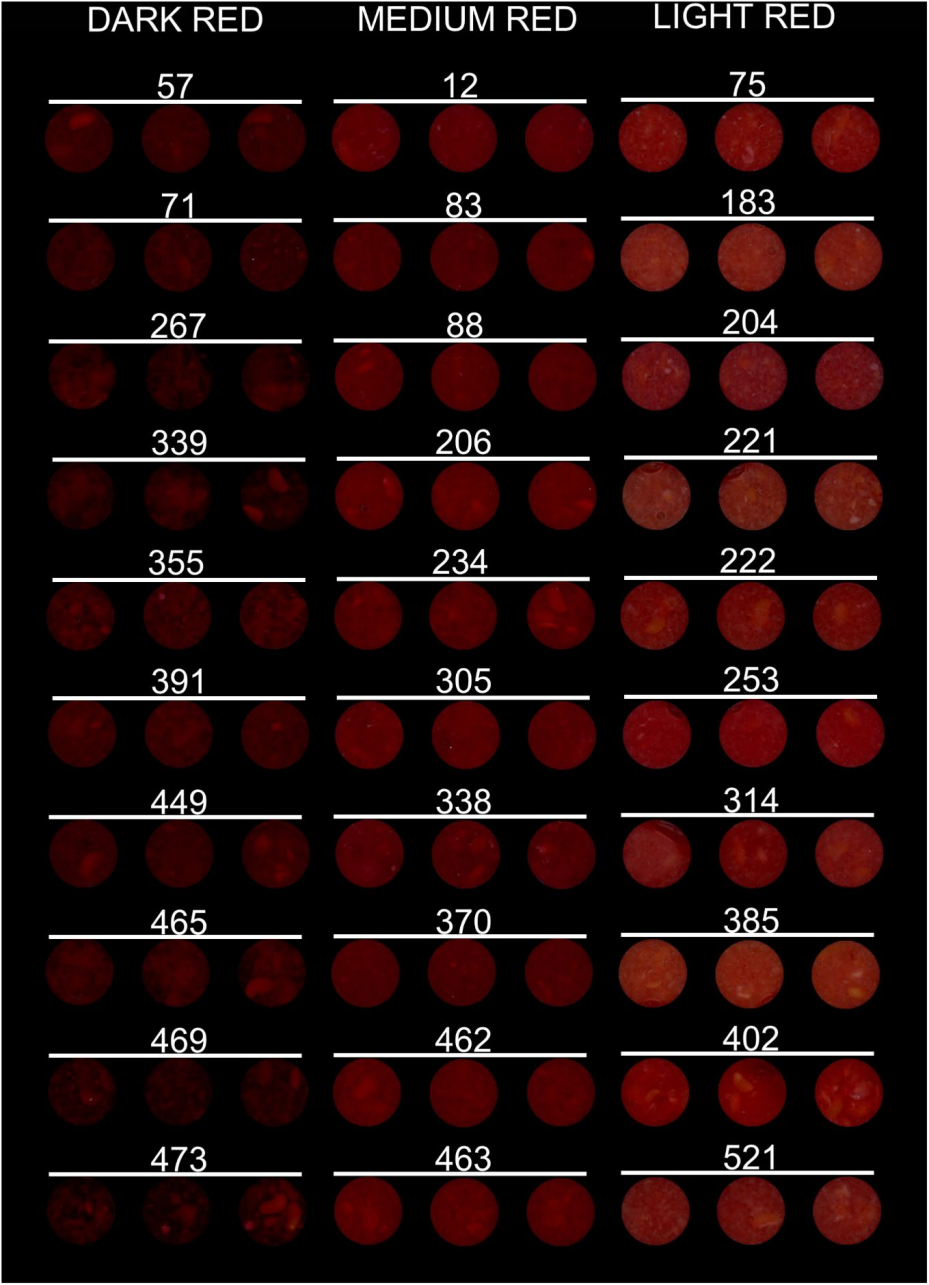
Dark, medium, and light red raspberry puree samples. The 30 raspberry puree samples (*n* = 3) were unanimously categorized by three operators as dark, medium, and light red. The numbers reported in the figure are the genotype IDs corresponding to each sample

To investigate potential improvements in total anthocyanin estimation using color parameters, the complete panel of 551 raspberry genotypes were grouped in three color categories – dark red, medium red, and light red – and correlation analyses between color coordinates and total anthocyanin content were repeated for each individual group. To define the different groups, three panelists visually scored images of 30 red raspberry puree samples and classified them as dark, medium, or light red, with ten raspberry samples assigned to each color category (Fig. 1).

**Fig. 2:**
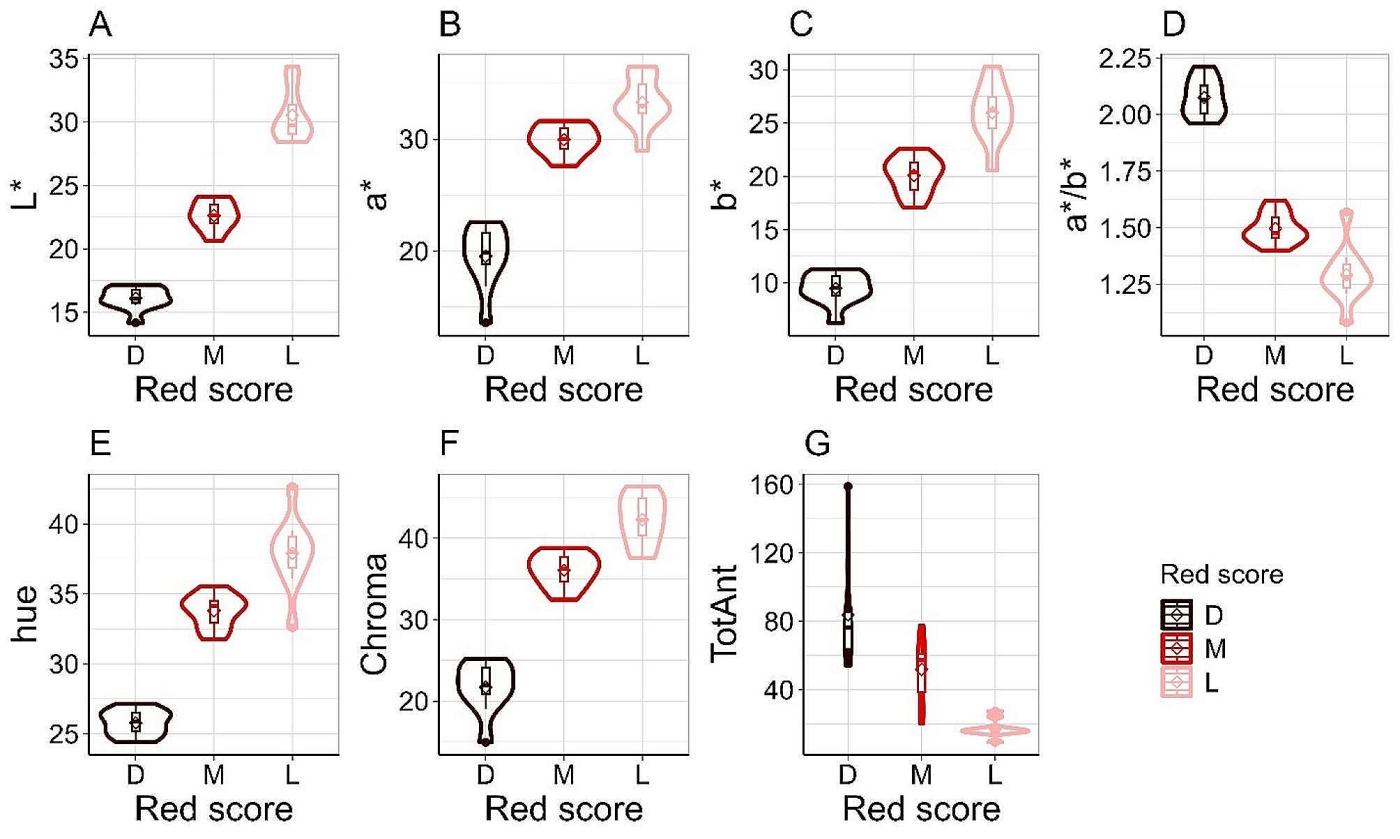
Relationship between red score (dark, medium, light), color parameters, and total anthocyanins. The violin plots show the association between assigned red scores – dark (D), medium (M), light (L) – color parameters (**A**–**F**), and total anthocyanins (**G**) of 30 genotypes (ten genotypes per color category)

The samples were empirically analyzed for the association between color perception (red score, i.e., dark, medium, or light), color parameters, and total anthocyanin content (Fig. 2). There was an almost perfect match between the color coordinate L* and the visually assigned red score: all samples regarded as dark red clustered together at low L* values; on the other hand, samples defined as light red all clustered together at high L* values (Fig. 2A). Samples scored as medium had intermediate L* values between light and dark samples (Fig. 2A). The a*/b* ratio also matched the color score, except for a slight overlap between the medium and light red color categories (Fig. 2D). There was no overlap of total anthocyanin values between dark and light red genotypes, while some of the medium red genotypes had anthocyanin values that overlapped with anthocyanin values of light or dark red genotypes (Fig. 2G).

**Fig. 3:**
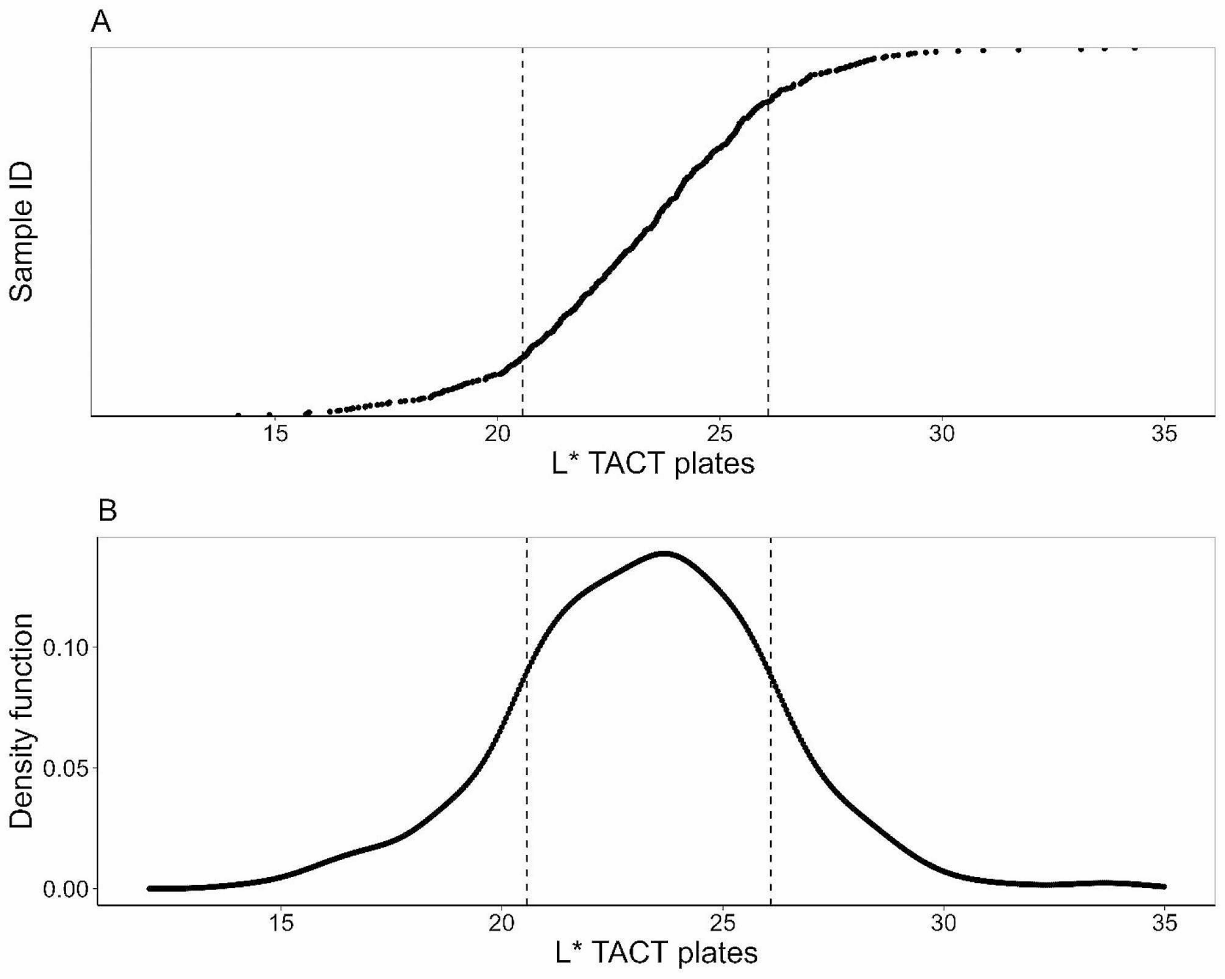
Distribution (**A**) and gaussian fit (**B**) of L* values for 551 raspberry genotypes. The dashed lines represent the inflection points at 20.56 and 26.08

Given the close association between red color perception and L* values, the grouping of the 551 genotypes was attained with kernel density on the values of color parameter L*. The resulting density function data were fitted with a gaussian curve to determine the mean (23.32) and the standard deviation (2.76) of the distribution. The inflection points of the curve, calculated as mean ± standard deviation (20.56; 26.08), were arbitrarily considered threshold values to group genotypes in color categories dark (L* < 20.56), medium (20.56 ≤ L* ≤ 26.08), and light (L* > 26.08). According to this approach, 87 genotypes were considered dark red, 383 medium red, and 81 light red. Figure 3A shows the distribution of all the genotypes ordered according to L* values and Fig. 3B depicts the gaussian distribution fit to the data.

### Statistical analysis

All statistical analyses were conducted using R version 4.3.3 [39]. Packages and functions are reported as ‘package name: function name’. Correlations heatmaps were obtained with corrplot: corrplot [40] and all the other plots were created by combining functions from packages ‘ggplot2’ [41], ‘ggpubr’ [42], and ‘ggpmisc’ [43]. Summary statistics – mean, standard deviation, and relative standard deviation – of color parameters L*, a*, b*, a*/b, hue, and Chroma were computed for the 126 samples analyzed with the colorimeter and TACT using Petri dishes and 96-well plates (Additional file 7: Table S3). Simple linear regression models were fit with the base R ‘lm’ function to compare color coordinates obtained with the three methodologies and were considered significant at *p* < 0.05. Assumptions of linearity, normality, and homoscedasticity were verified with diagnostic plots from the base R ‘plot’ function. Simple linear correlations using Pearson’s correlation method were performed to assess the relationship between color coordinates and total anthocyanin content and were considered significant at *p* < 0.05. Assumptions of linearity and normality were tested with GGally: pairs [44]. Grouping of the complete panel of 551 samples through kernel density was performed with stats: density. The gaussian fit on the density function data was attained with stats: nls. The R scripts used to analyze the data are provided as an R markdown file in the Supplementary Information (Additional file 8).

## Results

### Comparisons between color measurement methods

**Fig. 4:**
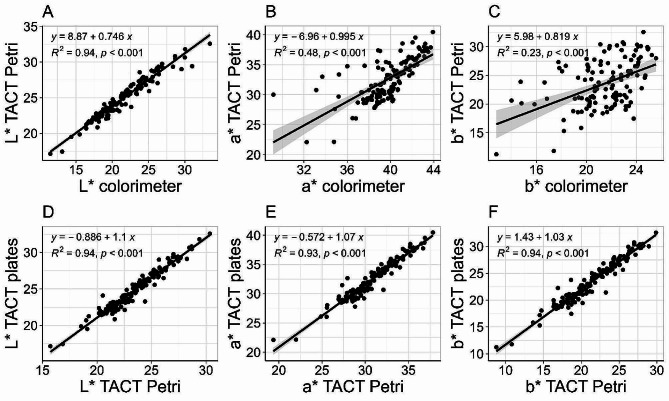
Comparisons between L*, a*, and b* measured with three methods. The scatterplots compare color parameters L*, a*, and b* collected using the colorimeter and TACT Petri dishes (**A**–**C**), and color parameters L*, a*, and b* collected using TACT Petri dishes and TACT plates (**D**–**F**). Simple linear regressions (*R*^*2*^) were fit to the data and were considered significant at *p* < 0.05

The regression model for parameter L*collected with the colorimeter and with TACT Petri dishes was significant (*p* < 0.001) and revealed strong consistency between the two methods (*R*^*2*^ = 0.94) (Fig. 4). Regression models between these two methods were also significant for a* and b* (*p* < 0.001), with *R*^*2*^ values indicating low to moderate levels of explained variance (*R*^*2*^ = 0.48 for a* and *R*^*2*^ = 0.23 for b*). When comparing measurements conducted with TACT using Petri dishes and plates, the regressions between each pair of color coordinates were significant (*p* < 0.001) and demonstrated strong consistency (*R*^*2*^ = 0.94 for L* and b*, *R*^*2*^ = 0.93 for a*). Regression analyses between methodologies were also conducted for the derived parameters a*/b*, hue, and Chroma (Additional file 9: Fig. S5).

### Correlation analysis between total anthocyanin and color parameters measured with different methods

#### Panel of 126 genotypes

**Fig. 5:**
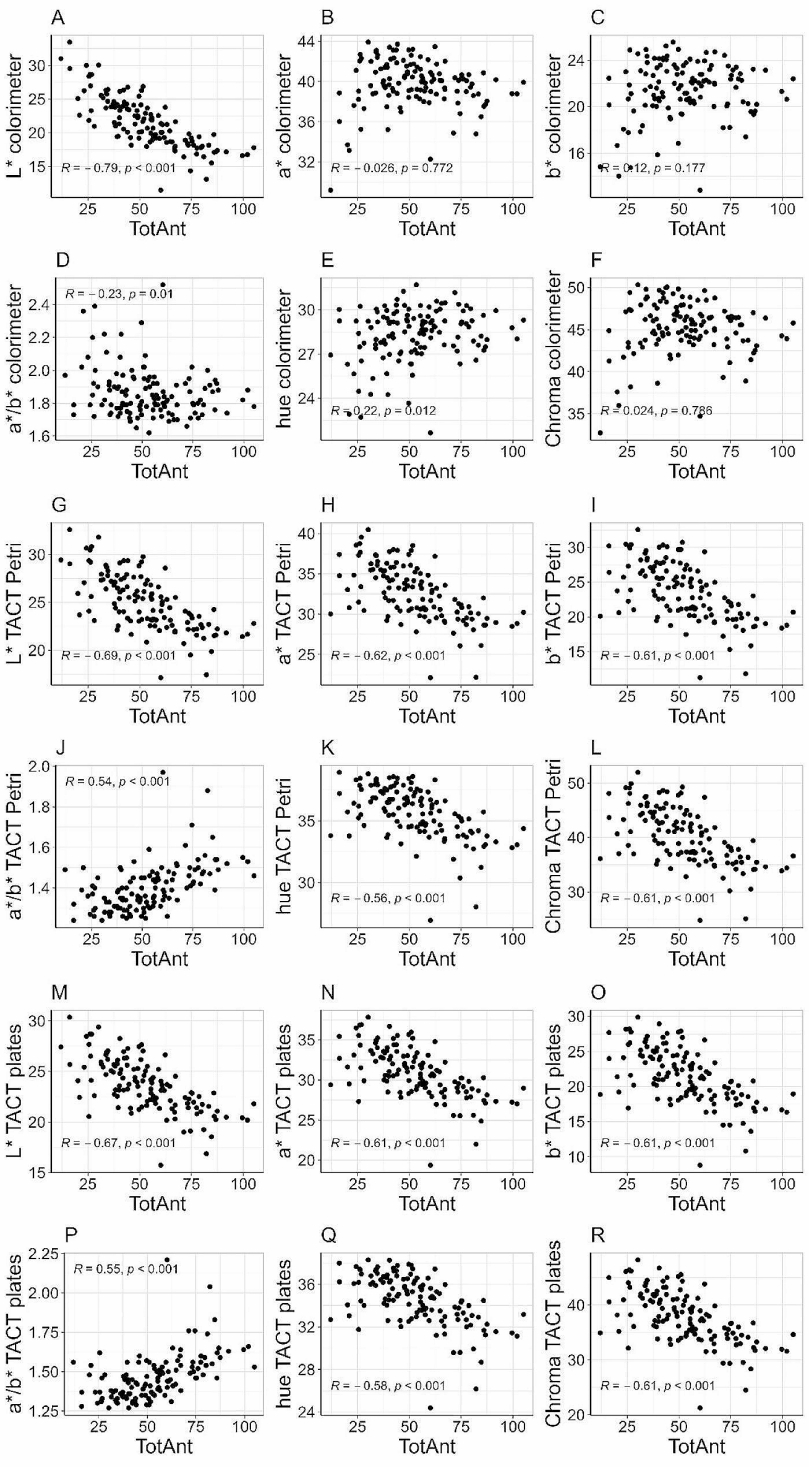
Correlation analysis between total anthocyanins and color parameters collected with three methods. The scatterplots compare total anthocyanins (TotAnt) with color parameters L*, a*, b*, a*/b*, hue, and Chroma for 126 red raspberry genotypes. Color coordinates were obtained with three methods, i.e., colorimeter (**A**–**F**), TACT using Petri dishes (**G**–**L**), and TACT using 96-well plates (**M**–**R**). Correlations were conducted with Pearson’s method (*R*) and were considered significant at *p* < 0.05

Significance and strength of correlations between total anthocyanins and color coordinates differed according to the methods used for color measurement (Fig. 5). Considering the color parameters measured with the colorimeter, total anthocyanins had a moderate negative correlation (*R* = − 0.79, *p* < 0.001) with L* (Fig. 5A), a weak negative correlation (*R* = − 0.23, *p* < 0.010) with the a*/b* ratio (Fig. 5D), and a weak positive correlation (*R* = 0.22, *p* < 0.012) with hue (Fig. 5E). The correlations of total anthocyanins with a*, b*, and Chroma measured with the colorimeter were not statistically significant (*p* ≥ 0.05). Total anthocyanins shared very similar correlation patterns with color parameters obtained with TACT Petri dishes and TACT plates (Fig. 5G and R). Total anthocyanins were negatively correlated with L*, and the correlation was moderate for both Petri dishes (*R* = − 0.69, *p* < 0.001) and for plates (*R* = − 0.67, *p* < 0.001). Notably, when using TACT significant correlations were found between total anthocyanins and a* and b* (Fig. 5H, I, N and O). Both the parameters were inversely correlated to total anthocyanins when using Petri dishes (*R* = − 062 for a* and *R* = − 0.61 for b*) and plates (*R* = − 0.61 for a* and *R* = − 0.61 for b*). Similar to what observed for a*/b* and hue measured with the colorimeter, significant correlations were detected between total anthocyanins and a*/b* and hue measured with the TACT methods (Fig. 5J, K, P and Q). However, while for the colorimeter the correlations were negative for a*/b* and positive for hue, these trends were reversed when using TACT methods: total anthocyanins positively correlated with a*/b* when using TACT Petri dishes (*R* = 0.54) and TACT plates (*R* = 0.55), and total anthocyanins were negatively associated with hue when using TACT Petri dishes (*R* = − 0.56) and TACT plates (*R* = − 0.58). It was also evident that the strength of the correlations of TACT methods considerably improved compared to the colorimeter measurements. Conversely to the colorimeter, the TACT methods found a significant correlation between total anthocyanins and Chroma (Fig. 5L and R), with an inverse and moderately strong relationship (*R* = − 0.61 for both Petri dishes and plates).

#### Panel of 551 genotypes

**Fig. 6:**
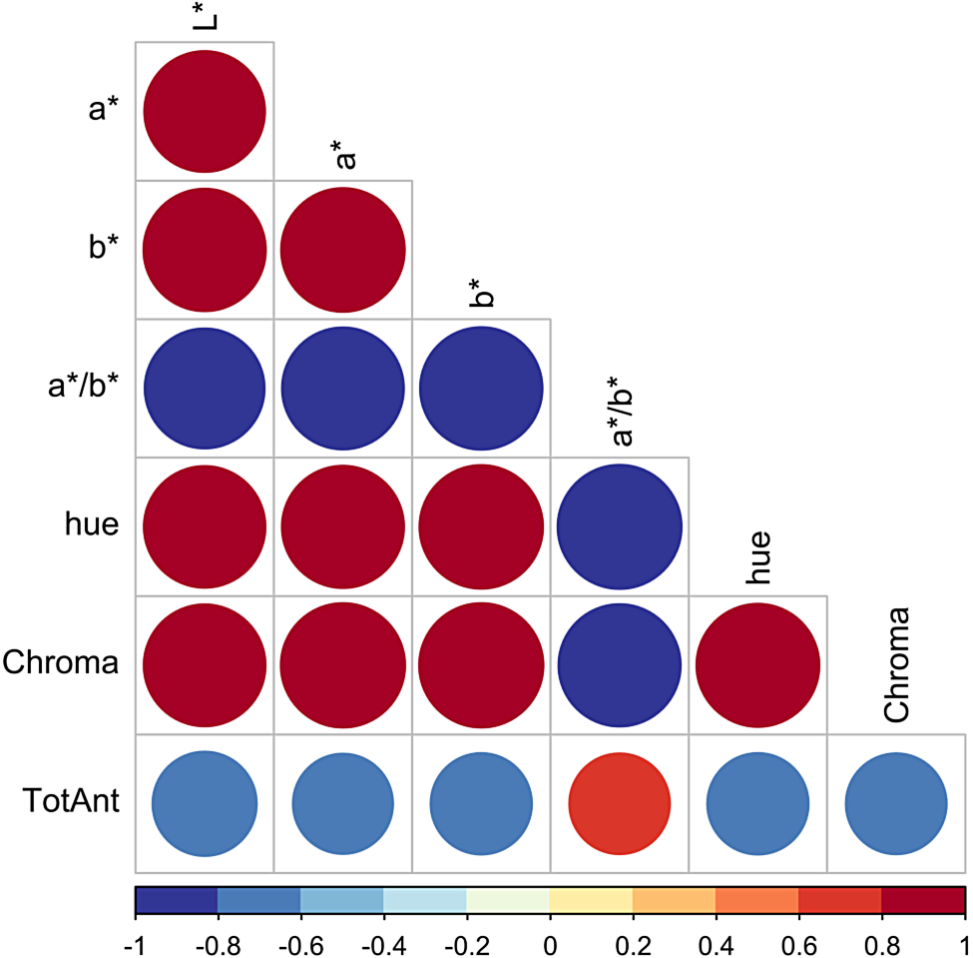
Correlation analysis between color parameters and total anthocyanins (TotAnt) for 551 raspberry genotypes. The correlation heatmap compares total anthocyanins (TotAnt) with color parameters L*, a*, b*, a*/b*, hue, and Chroma for 551 red raspberry genotypes. Color coordinates were obtained with TACT using 96-well plates. Blue and red colors represent negative and positive correlations, respectively, and the shade and size of the circles represent the strength of the correlations (the darker the shade and the larger the circle the stronger the correlation). Correlations were conducted with Pearson’s method (*R*) and were considered significant at *p* < 0.05

Correlations between total anthocyanins and color parameters obtained with TACT plates were further studied in a larger panel of 551 genotypes. Results of this analysis confirmed observations from the smaller panel of 126 genotypes: all correlations between total anthocyanins and color parameters were significant (*p* < 0.001) and negative, except for a*/b*, which had a positive correlation with total anthocyanins (*R* = 0.66, Fig. 6). The strengths of correlations observed for the larger panel of genotypes were similar to those of the smaller panel, and L* consistently displayed the strongest relationship with total anthocyanins (*R* = − 0.70, Fig. 6).

### Correlation analysis between total anthocyanins and color parameters within color groups

**Fig. 7:**
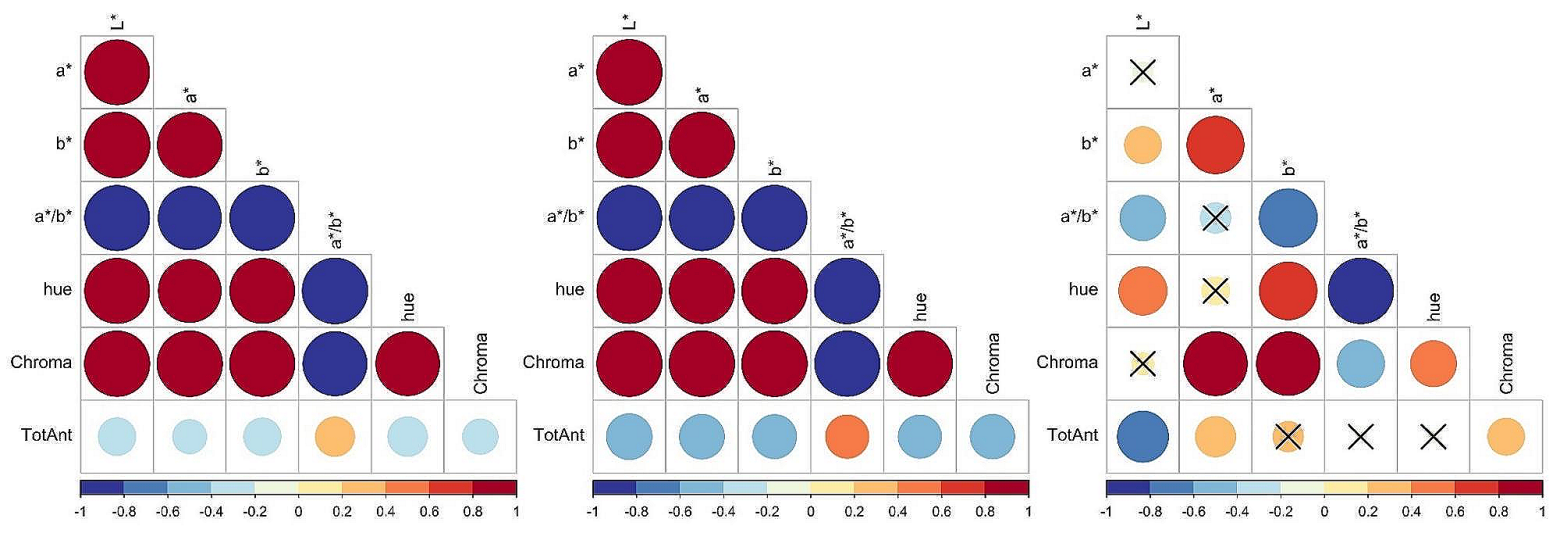
Correlation analysis between total anthocyanins (TotAnt) and color parameters for genotypes with different color intensities. The correlation heatmaps compare total anthocyanins (TotAnt) with color parameters L*, a*, b*, a*/b*, hue, and Chroma for raspberry genotypes classified in three color categories – dark (**A**), medium (**B**), and light (**C**) – based on L* values as in Fig. 3. Results were obtained using 87, 383, and 81 genotypes for the dark, medium, and light red categories, respectively. Blue and red colors represent negative and positive correlations, respectively, and the shade and size of the circles represent the strength of the correlations (the darker the shade and the larger the circle the stronger the correlation). Correlations were conducted with Pearson’s method (*R*) and were considered significant at *p* < 0.05. Non-significant correlations (*p* ≥ 0.05) are marked with a cross (×)

Correlation trends of dark and medium red genotypes reflected the trends observed for the pooled genotypes: all correlations were statistically significant (*p* < 0.05) and all color parameters were negatively correlated with total anthocyanin content, except for the a*/b* ratio (*R* = 0.36, *p* < 0.001 for the dark category and *R* = 0.44, *p* < 0.001 for the medium category) (Fig. 7A and B). However, correlations did not improve within individual groups. For dark red samples, total anthocyanins showed the strongest correlation with hue (*R* = − 0.37, *p* < 0.001), followed by a*/b* (*R* = 0.36, *p* < 0.001), and L* (*R* = − 0.33, *p* < 0.001) (Fig. 7A). In the medium red category, total anthocyanins showed the strongest correlation with L* (*R* = − 0.49, *p* < 0.001), followed by a* (*R* = − 0.47, *p* < 0.001), and Chroma (*R* = − 0.46, *p* < 0.001). The results for light red genotypes were different than the other color categories. Except for L* (*R* = − 0.61, *p* < 0.001), a* (*R* = 0.38, *p* < 0.001), and Chroma (*R* = 0.32, *p* < 0.01), the other correlations between total anthocyanins and color parameters were not significant (Fig. 7C). Moreover, for light red genotypes the relationship between total anthocyanins and L* had a similar strength as observed when combining all genotypes (*R* = − 0.70, Fig. 6).

## Discussion

Phenotyping of plant traits is widely regarded as the most challenging step in the plant breeding pipeline [45, 46], partly because of the large number of individuals commonly screened and the lack of high-throughput phenotyping techniques. The present study tested the use of the digital phenotyping software module Tomato Analyzer Color Test (TACT), originally developed for fresh tomato but later applied to other fresh fruits, to conduct high-throughput assessment of red raspberry puree color and to predict total anthocyanin content.

The significant and consistent regression models found between the measurement of L* through the colorimeter and TACT Petri dishes suggests that TACT Petri dishes could be a valid substitute of the colorimeter for the assessment of L*. Instead, the low to moderate consistency observed between a* and b* measured with the colorimeter and TACT Petri dishes indicate the two methods might not be interchangeable for the measurement of a* and b*. When considering color parameters obtained with TACT Petri dishes and TACT plates, the regressions were significant for all color parameters L*, a*, b*, and *R*^*2*^ was close to 1, clearly pointing out that the 96-well plates are a valid substitute for Petri dishes to conduct high-throughput color measurement of red raspberry puree. Based on our experiments, the adoption of the TACT plates method would enable faster color analysis than the TACT Petri dishes method (2–3 times faster) and the colorimeter (3–4 times faster). Additionally, the use of 96-well plates would reduce the amount of fruit puree and laboratory supplies needed for color analysis compared to 3.5-cm Petri dishes.

Regarding the correlations of color parameters with total anthocyanins, the results clearly depicted different performances of the colorimeter and TACT. While significant relationships were established between all color parameters (L*, a*, b*, a*/b*, hue, and Chroma) and total anthocyanin content for TACT-based methods, the colorimeter failed to detect significant correlations between parameters a*, b*, and Chroma and total anthocyanins. The TACT-based methods consistently recorded inverse relationships between color parameters and total anthocyanin content, except for a*/b*, whose relationship with anthocyanins was positive. Instead, the colorimeter found an inverse correlation between a*/b* and total anthocyanins, and a positive correlation between hue and total anthocyanins. The results obtained using TACT-based methods are in agreement with Moore [19] and Palonen and Weber [27], who also reported significant correlations and analogous trends between color parameters (measured with colorimeters) and total anthocyanins in red raspberry fruit. Instead, Mazur, Nes [16] only found a significant and moderate correlation for parameter L* (*R* = − 0.61). The results provided by TACT are consistent with a darkening of fruit color as total anthocyanin content increases. Lower L* values correspond to darker color intensities, lower a* and b* are indicative of color shifts from red to green and from yellow to blue, respectively, lower hue and Chroma reflect a more red/bluish color and a decrease in color intensity, respectively, and higher a*/b* reflects darker coloration. Overall, the methods based on TACT appear to be better than the colorimeter at detecting color discrepancies between red raspberry puree samples.

Moore [19] and Palonen and Weber [27] observed that in red raspberry the strongest correlations with total anthocyanins were established with a*/b* and hue. In this study, when comparing the three methods, the strongest correlation coefficients were found for the parameter L*. The negative trend and magnitude of correlation between total anthocyanins and L* were very similar between TACT methods (*R* = − 0.69 for TACT Petri dishes and *R* = − 0.67 for TACT plates), meaning that color parameter L* obtained with 96-well plates could approximate anthocyanin content as accurately as color parameters recorded with Petri dishes. The colorimeter scored the strongest coefficient for L* (*R* = − 0.79), but the coefficients observed for L* across all methods were within the range of moderately strong correlations. Correlation analysis using values from 551 genotypes measured with TACT plates confirmed the trends and strengths of correlations between color parameters and anthocyanins, including the fairly strong relationship between L* and total anthocyanins (*R* = − 0.70). Different interpretations on the predictability of total anthocyanins in red raspberry based on color parameters have been found in the literature. Moore [19] argued that both a*/b* (*R* = 0.73, *R*^*2*^ = 0.53) and hue (*R* = − 0.72) could be used as predictors of anthocyanin concentration in red raspberry fruit. Instead, Palonen and Weber [27] stated that despite finding good correlations between anthocyanin concentration and a*/b and hue (respectively *R* = 0.764 and *R* = − 0.738), they could not use surface color values to directly estimate anthocyanin concentration because differences in the glossiness of the fruit surface may bias the prediction of total anthocyanins inside the berries. Previous studies have highlighted the contribution of waxes and trichomes to surface color of various fruits (blueberry [47]; cucumber [48]; peach [49]; tomato [50]). The use of puree instead of fresh fruit might represent a way to exclude or reduce the confounding effect of waxes and trichomes on raspberry fruit color. However, from a breeding perspective the correlations found in this study using TACT may not be sufficient to build models that confidently estimate total anthocyanins in red raspberry puree. Taking the example of L*, the parameter showing the strongest correlation with anthocyanins, a correlation coefficient of − 0.70 corresponds to a coefficient of determination (*R*^*2*^) of 0.49, meaning that a simple linear regression model built on L* would explain about 49% of the variation in total anthocyanins. Panthee, Perkins-Veazie [33] studied the correlation between color parameters and lycopene content of tomatoes and observed that some color parameters displayed better correlations with lycopene content when tomatoes were grouped in color categories (dark red, red, and pink). They leveraged multiple regression analysis to develop models to predict lycopene content in tomatoes and obtained an equation that explained almost 99% of the variation in pink tomatoes. Here, correlation analyses within individual color groups found weaker correlations than the approach that pooled all samples together. Interestingly, the correlation coefficients between L* and total anthocyanins became stronger moving from darker to lighter red genotypes. Panthee, Perkins-Veazie [33] obtained increasing *R*^*2*^ going from dark red to red and pink tomatoes. The weaker correlations observed in the present study for the dark and medium red categories might be attributed to a saturation effect of anthocyanin concentration, for which an increase in total anthocyanins or in specific anthocyanins conferring a darker color would not cause a linear response of the color parameters beyond a certain threshold. Aside from saturation, more factors could justify the poor performance of color parameters in estimating total anthocyanins. The final color of red raspberries is not only dependent on the total amount of anthocyanins, but also on the presence/absence and relative abundance of specific anthocyanins [51]. Anthocyanin profiles have been reported to differ according to the raspberry genotypes considered [16, 52]. Mazur, Nes [16] reported that relative concentrations of cyanidin-3-sophoroside could vary from 47% in ‘Veten’ to 79% in ‘Malling Hestia’, those of cyanidin-3-(2g-glucosylrutinoside) from 1% in ‘Malling Hestia’ to 31% in ‘RU004 04095’, and those of cyanidin-3-glucoside from 7% in ‘RU974 07002’ to 24% in ‘Glen Magna’. Still, none of the color parameters (L*, hue, Chroma) assessed in the same study showed a strong correlation with individual anthocyanins. The estimation of anthocyanins through color parameters is further complicated by the fact that anthocyanin structure, and consequently the absorption spectrum, is affected by the pH of the surrounding environment (the vacuole): anthocyanins turn red-orange at pH < 3, violet between pH 6–7, blue between pH 7–8, and blue-green at pH > 11 [53]. Hence, even in the presence of similar relative abundance and total concentrations, different pH values may trigger different pigmentations and therefore contribute to weakening the association between fruit color and total anthocyanin content. Lastly, the interaction of anthocyanins with certain colorless compounds, a phenomenon known as co-pigmentation, can reinforce anthocyanin pigmentation and this could further increase the discrepancy between observed fruit color and total anthocyanins. Several different compounds are thought to be involved in co-pigmentation, including other anthocyanins, flavonoids, polysaccharides, alkaloids, amino acids, organic acids, nucleotides, and metals [54]. To the authors’ knowledge, no studies are available about co-pigmentation in red raspberry. Besides anthocyanins, carotenoids are other important pigments found in red raspberries, however carotenoids’ contribution to red raspberry fruit color should be masked by anthocyanins [10], suggesting that carotenoids may not interfere with the relationship between total anthocyanins and the red pigmentation of raspberry fruit.

## Conclusions

The regression analyses of raspberry puree color parameters collected with the colorimeter and TACT Petri dishes suggests that the two techniques are interchangeable for the assessment of L*, but not a* and b*. The consistency observed between color coordinates obtained with TACT Petri dishes and TACT plates indicates that 96-well plates are a valid substitute of Petri dishes and their use in the color measurement protocol would significantly increase the throughput of the analysis. Color coordinates obtained with TACT-based methods exhibited stronger relationships with total anthocyanins than color coordinates collected with the colorimeter, and all color coordinates pointed toward a shift to darker colors, with higher green and blue components, when anthocyanin content increased. Taken together, these results imply that TACT better detects color changes than the colorimeter, cementing the validity of the high-throughput methodology here developed. Nevertheless, the correlations observed were not strong enough to produce models for the prediction of total anthocyanin content using color coordinates. Correlation analyses on color groups did not find better correlations than the analysis conducted on the full panel of genotypes. The complexity of red raspberry fruit color might prevent color coordinates alone to reliably predict total anthocyanin content and therefore screening of total anthocyanins in red raspberry should still rely on wet lab extraction and quantification techniques.

### Electronic supplementary material

Below is the link to the electronic supplementary material.

**Supplementary Material 1**: Additional Figures and Tables

**Supplementary Material 2**: R Scripts

## Data Availability

The datasets used and/or analyzed during the current study are available from the corresponding author on reasonable request.
