# Supplementary material for: High-throughput color determination of red raspberry puree and correlation of color parameters with total anthocyanins
Source: Plant Methods. 2024 May 30;20:78. doi: 10.1186/s13007-024-01197-0 (PMC11137939; doi:10.1186/s13007-024-01197-0)
Supplement: Supplementary file 1 — Supplementary Material 1: Additional Figures and Tables [file 13007_2024_1197_MOESM1_ESM.docx]

**Supplementary Information**


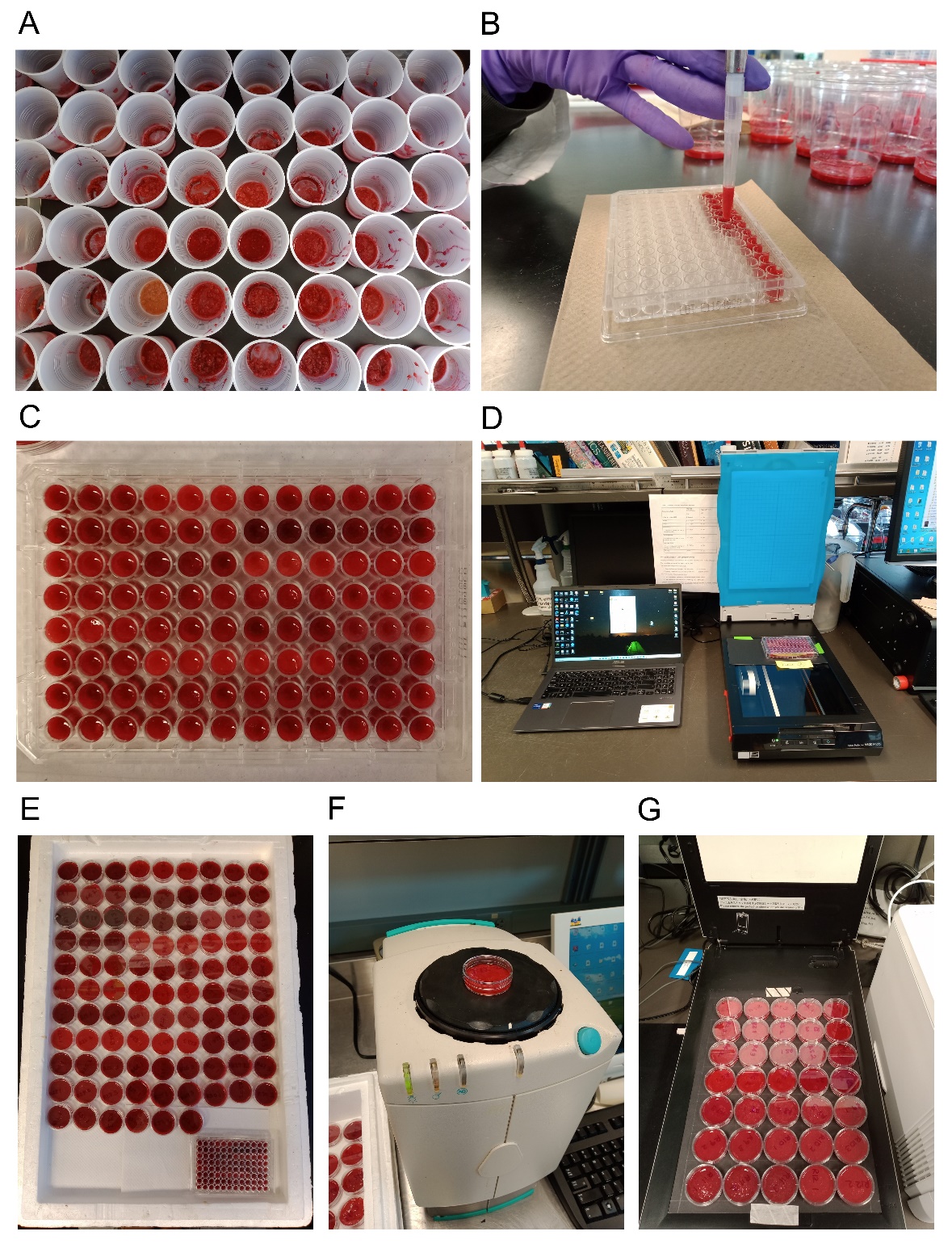


**Additional file 1: Fig. S1.** Experimental setup for color analyses with the colorimeter and Tomato Analyzer. Raspberry puree in plastic cups (**A**), plate preparation (**B**), finished plate (**C**), plate on the scanner for image acquisition (**D**), Petri dishes and plate for the same set of samples (**E**), Petri dish on the colorimeter sensor eye (**F**), Petri dishes on the scanner for image acquisition (**G**).

**
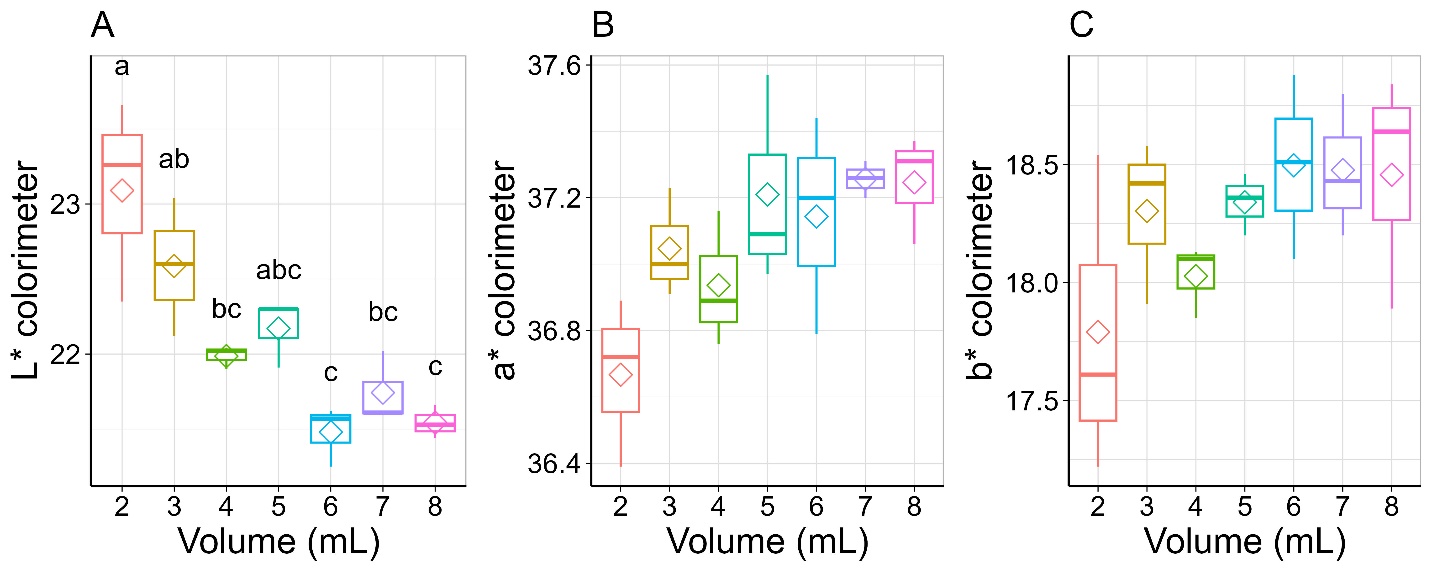
**

**Additional file 2: Fig. S2**. The effect of seven puree volumes on L*, a*, and b* values measured with the colorimeter. Seven puree volumes (n = 3) were tested to decide how much puree to use for color measurement with a colorimeter, to optimize the reproducibility of the measurement and the amount of sample material. The boxplots summarize the data for color coordinates L* (**A**), a* (**B**), and b* (**C**). ANOVA was conducted to test whether different volumes affected color parameters, and significance was determined at *p* < 0.05. Mean comparison was conducted with Tukey’s HSD test. Different letters denote significant differences.

**
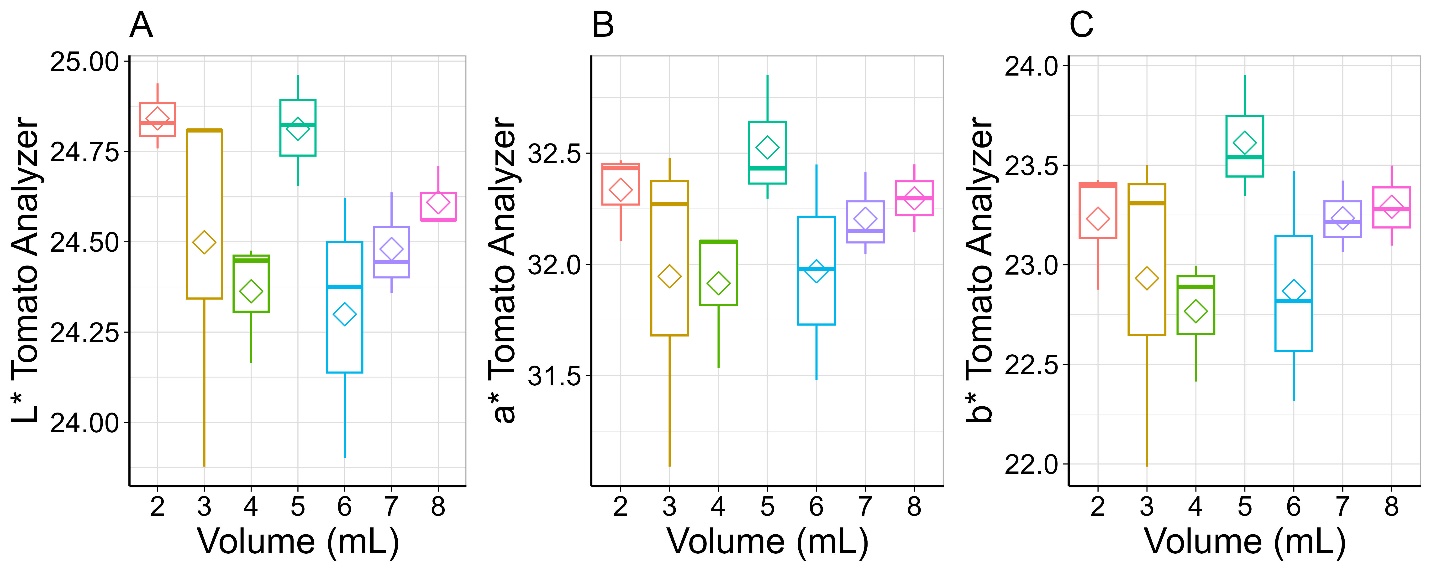
**

**Additional file 3: Fig.** **S3**. The effect of seven puree volumes on L*, a*, and b* values measured with TACT. Seven puree volumes (n = 3) were tested to decide how much puree to use for color measurement with TACT, to optimize the reproducibility of the measurement and the amount of sample material. The boxplots summarize the data for color coordinates L* (**A**), a* (**B**), and b* (**C**). ANOVA was conducted to test whether different volumes affected color parameters, and significance was determined at *p* < 0.05. Mean comparison was conducted with Tukey’s HSD test.

**Additional file 4: Table S1.** Descriptive statistics of L*, a*, and b* measured with the colorimeter.

|  | **Mean** | | | **Standard deviation** | | | **Relative standard deviation** | | |
| --- | --- | --- | --- | --- | --- | --- | --- | --- | --- |
| **Volume (mL)** | L* | a* | b* | L* | a* | b* | L* | a* | b* |
| 2 | 23.09 a | 36.67 | 17.79 | 0.67 | 0.25 | 0.68 | 2.91 | 0.69 | 3.81 |
| 3 | 22.59 ab | 37.05 | 18.30 | 0.46 | 0.17 | 0.35 | 2.04 | 0.45 | 1.91 |
| 4 | 21.99 bc | 36.94 | 18.03 | 0.08 | 0.20 | 0.15 | 0.34 | 0.55 | 0.85 |
| 5 | 22.17 abc | 37.21 | 18.34 | 0.23 | 0.32 | 0.13 | 1.02 | 0.85 | 0.72 |
| 6 | 21.48 c | 37.14 | 18.50 | 0.20 | 0.33 | 0.39 | 0.93 | 0.88 | 2.11 |
| 7 | 21.74 bc | 37.26 | 18.48 | 0.24 | 0.06 | 0.30 | 1.10 | 0.15 | 1.64 |
| 8 | 21.54 c | 37.25 | 18.46 | 0.11 | 0.16 | 0.50 | 0.51 | 0.44 | 2.71 |

Mean, standard deviation, and relative standard deviation of color coordinates L*, a*, and b* were obtained with the colorimeter using seven different puree volumes (n = 3). ANOVA was conducted on the means to test whether different volumes affected color measurement and significance was determined at *p* < 0.05. Mean comparison was conducted with Tukey’s HSD test. Different letters next to the mean L* values denote significant differences.

**Additional file 5: Table S2.** Descriptive statistics of L*, a*, and b* measured with TACT.

|  | **Mean** | | | **Standard deviation** | | | **Relative standard deviation** | | |
| --- | --- | --- | --- | --- | --- | --- | --- | --- | --- |
| **Volume (mL)** | L* | a* | b* | L* | a* | b* | L* | a* | b* |
| 2 | 24.84 | 32.34 | 23.23 | 0.09 | 0.20 | 0.31 | 0.37 | 0.62 | 1.33 |
| 3 | 24.50 | 31.95 | 22.93 | 0.54 | 0.75 | 0.82 | 2.20 | 2.34 | 3.59 |
| 4 | 24.36 | 31.92 | 22.77 | 0.17 | 0.33 | 0.31 | 0.71 | 1.03 | 1.36 |
| 5 | 24.81 | 32.53 | 23.61 | 0.15 | 0.29 | 0.31 | 0.62 | 0.89 | 1.31 |
| 6 | 24.30 | 31.97 | 22.87 | 0.37 | 0.48 | 0.58 | 1.51 | 1.52 | 2.54 |
| 7 | 24.48 | 32.20 | 23.23 | 0.14 | 0.19 | 0.18 | 0.58 | 0.59 | 0.78 |
| 8 | 24.61 | 32.30 | 23.29 | 0.09 | 0.15 | 0.20 | 0.35 | 0.47 | 0.86 |

Mean, standard deviation, and relative standard deviation of color coordinates L*, a*, and b* obtained with TACT using seven different puree volumes (n = 3). ANOVA was conducted on the means to test whether different volumes affected color measurement and significance was determined at *p* < 0.05. Mean comparison was conducted with Tukey’s HSD test.

**
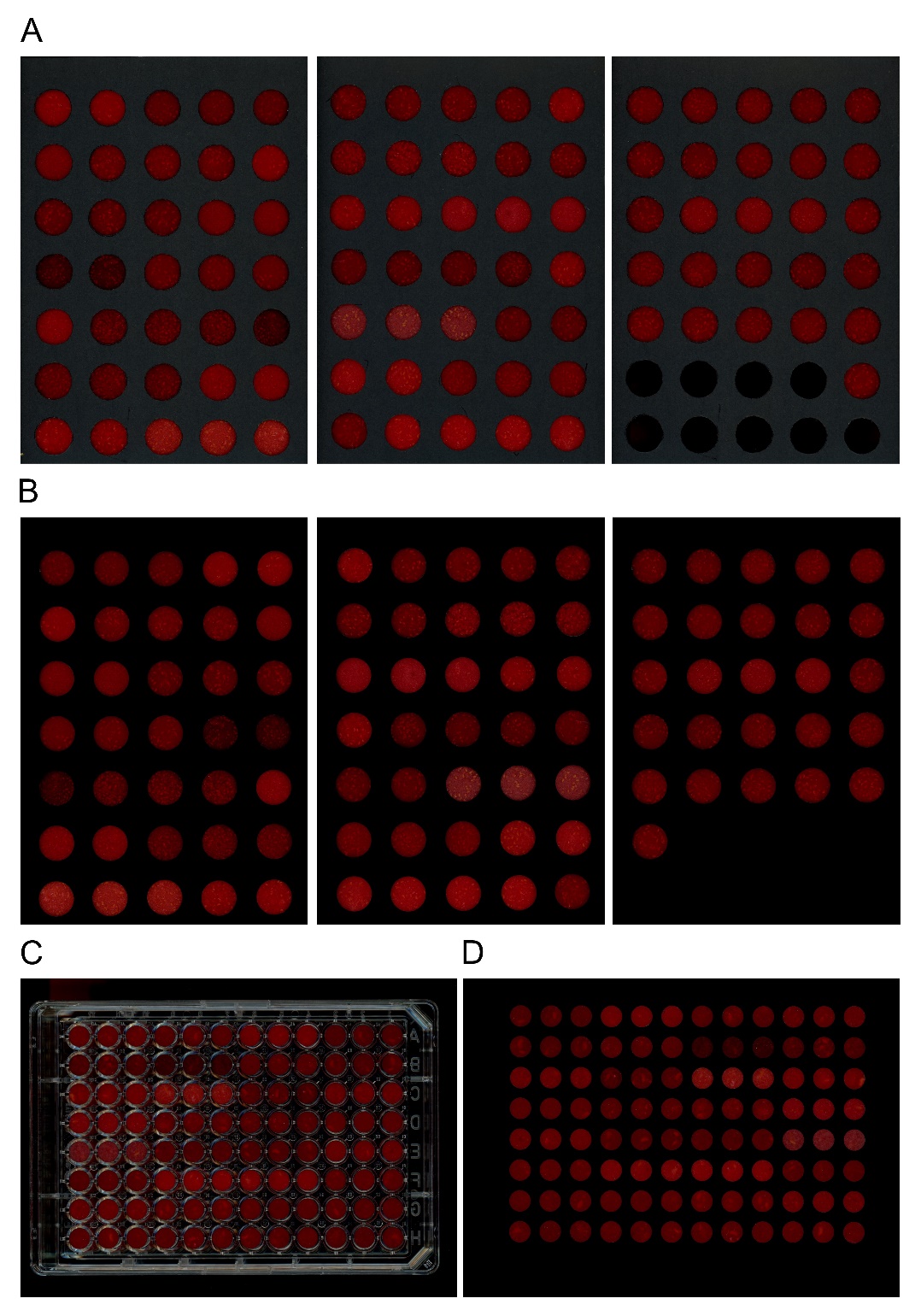
**

**Additional file 6: Fig. S4.** Original scans of Petri dishes (**A**) and of a 96-well plate (**C**, cropped from the original) and corresponding edited images (**B**, **D**). In the edited images the original background was replaced with a true black background.

**Additional file 7: Table** **S3**. Descriptive statistics of color parameters L*, a*, b*, a*/b*, hue, and Chroma measured with three methods.

|  | **Colorimeter** | | | | | | **TACT Petri dishes** | | | | | | **TACT plates** | | | | | |
| --- | --- | --- | --- | --- | --- | --- | --- | --- | --- | --- | --- | --- | --- | --- | --- | --- | --- | --- |
|  | L* | a* | b* | a*/b* | hue | Chroma | L* | a* | b* | a*/b* | hue | Chroma | L* | a* | b* | a*/b* | hue | Chroma |
| **MEAN** |  |  |  |  |  |  |  |  |  |  |  |  |  |  |  |  |  |  |
| Mean | 21.64 | 39.76 | 21.51 | 1.86 | 28.33 | 45.22 | 25.01 | 32.61 | 23.60 | 1.40 | 35.62 | 40.28 | 23.62 | 30.96 | 21.56 | 1.46 | 34.55 | 37.75 |
| Min | 11.48^71^ | 29.18^84^ | 12.81^71^ | 1.62^82^ | 21.65^71^ | 32.73^84^ | 17.14^71^ | 22.11^71^ | 11.23^71^ | 1.24^75,87^ | 26.93^71^ | 24.80^71^ | 15.73^71^ | 19.37^71^ | 8.79^71^ | 1.27^50,87^ | 24.39^71^ | 21.27^71^ |
| Max | 33.46^75^ | 43.92^87^ | 25.56^8^ | 2.52^71^ | 31.69^82^ | 50.32^87^ | 32.59^75^ | 40.48^87^ | 32.57^87^ | 1.97^71^ | 38.94^75^ | 51.96^87^ | 30.36^75^ | 37.83^87^ | 29.88^87^ | 2.21^71^ | 38.30^87^ | 48.21^87^ |
| **RSD** |  |  |  |  |  |  |  |  |  |  |  |  |  |  |  |  |  |  |
| Mean | 1.81 | 0.52 | 1.74 | 1.54 | 0.01 | 0.68 | 1.39 | 1.34 | 2.39 | 1.09 | 0.85 | 1.67 | 1.64 | 1.57 | 2.62 | 1.48 | 1.14 | 1.99 |
| Min | 0.09 | 0.03 | 0.11 | 0.00 | 0.00 | 0.04 | 0.06 | 0.12 | 0.08 | 0.00 | 0.04 | 0.11 | 0.22 | 0.18 | 0.08 | 0.00 | 0.10 | 0.28 |
| Max | 7.61 | 1.58 | 7.53 | 6.99 | 0.06 | 2.88 | 5.29 | 5.71 | 12.54 | 7.47 | 5.41 | 8.10 | 6.69 | 13.89 | 12.60 | 5.45 | 4.26 | 15.29 |

Mean, minimum (Min), and maximum (Max) values of means and relative standard deviations (RSD) of color parameters L*, a*, b*, a*/b*, hue, and Chroma of 126 puree samples (n = 3) measured with the colorimeter, TACT Petri dishes, and TACT plates. The numbers reported as underlined superscripts are the genotype IDs corresponding to those values.


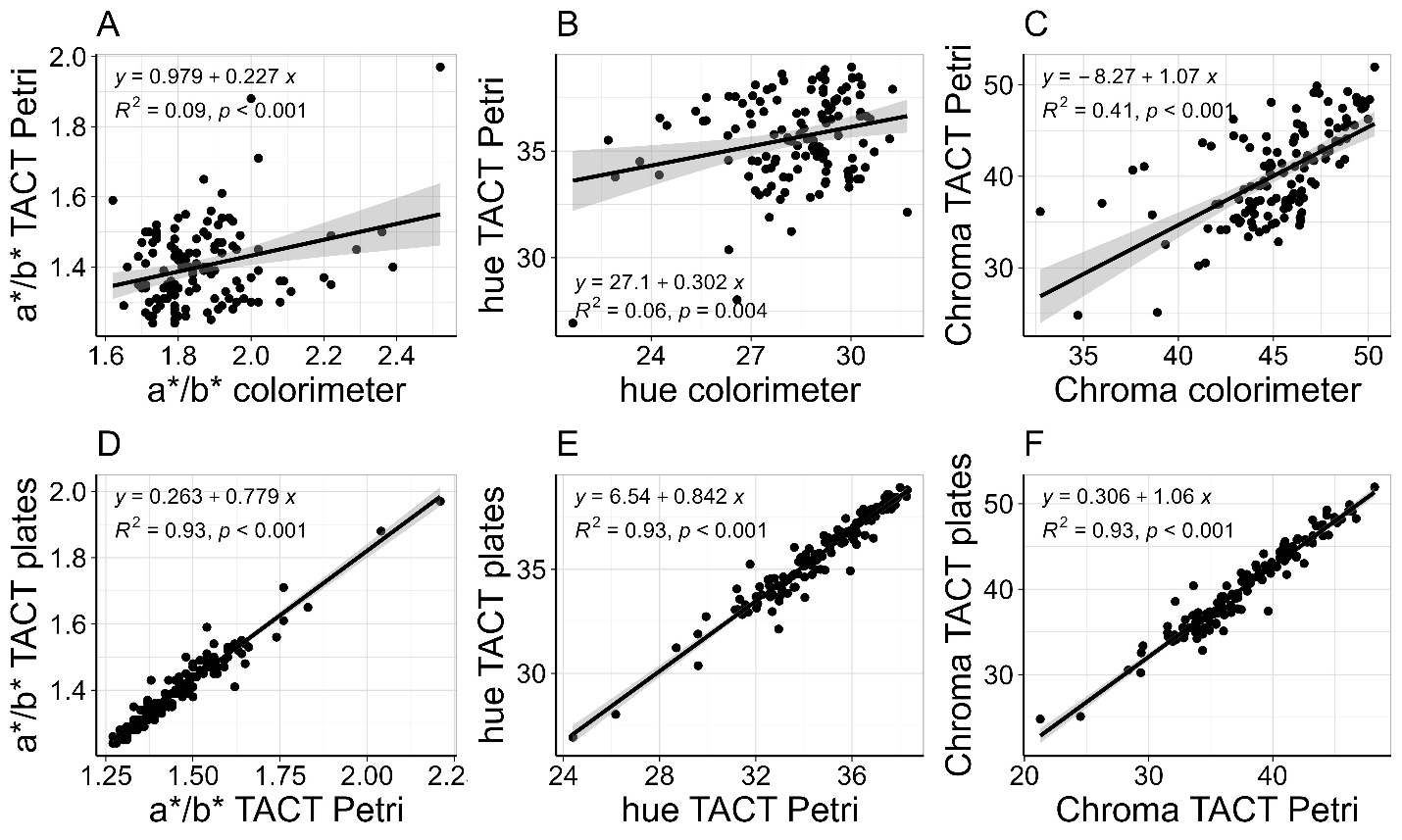


**Additional file 9: Fig. S5.** Comparisons between a*/b*, hue, and Chroma measured with three methods. The scatterplots compare color parameters a*/b*, hue, and Chroma collected using the colorimeter and TACT Petri dishes (**A–C**), and color parameters a*/b*, hue, and Chroma collected using TACT Petri dishes and TACT plates (**D–F**). Simple linear regressions (*R^2^*) were fit to the data and were considered significant at *p* < 0.05.
